# Longitudinal Clinical Performance of the RNA-Based Aptima Human Papillomavirus (AHPV) Assay in Comparison to the DNA-Based Hybrid Capture 2 HPV Test in Two Consecutive Screening Rounds with a 6-Year Interval in Germany

**DOI:** 10.1128/JCM.01177-18

**Published:** 2019-01-02

**Authors:** Thomas Iftner, Klaus-Joachim Neis, Alejandra Castanon, Rebecca Landy, Barbara Holz, Astrid Woll-Herrmann, Angelika Iftner, Annette Staebler, Diethelm Wallwiener, Claus Hann von Weyhern, Felix Neis, Juliane Haedicke-Jarboui, Peter Martus, Sara Brucker, Melanie Henes, Peter Sasieni

**Affiliations:** a Institute of Medical Virology and Epidemiology of Viral Diseases, University Hospital Tübingen, Tübingen, Germany; b Frauenärzte am Staden, Saarbrücken, Germany; c Centre for Cancer Prevention, Queen Mary University of London, London, United Kingdom; d Cancer Prevention Group, School of Cancer & Pharmaceutical Sciences, Faculty of Life Sciences & Medicine, King’s College London, London, United Kingdom; e Department of Pathology and Neuropathology, University Hospital Tübingen, Tübingen, Germany; f Department of Gynaecology and Obstetrics, University Hospital Tübingen, Tübingen, Germany; g Medical Faculty Tübingen, Institute for Clinical Epidemiology and Applied Biometry, Tübingen, Germany; Memorial Sloan Kettering Cancer Center

**Keywords:** Aptima HPV, E6/E7 mRNA, cervical cancer screening, cervical intraepithelial neoplasia

## Abstract

Longitudinal data on the E6/E7 mRNA-based Aptima human papillomavirus (AHPV) assay exceeding three years in comparison to the gold standard Digene Hybrid Capture 2 (HC2) test are not available. We previously reported the cross-sectional data of the German AHPV Screening Trial (GAST) in which 10,040 women were recruited and tested by liquid-based cytology, the HC2 assay, and the AHPV assay. Four hundred eleven test-positive women were followed for up to six years. In addition, 3,295 triple-negative women were screened after a median time of six years. Overall, 28 high-grade cervical intraepithelial neoplasia (CIN3) cases were detected. The absolute risk of developing high-risk HPV-positive CIN3+ over six years among those women that tested negative at baseline was 2.2 (95% confidence interval [95% CI], 1.0 to 4.9) and 3.1 (95% CI, 1.7 to 5.7) per 1,000 women screened by the HC2 and the AHPV tests; the additional risk for those with AHPV-negative compared with HC2-negative results was 0.9 (95% CI, −0.2 to 2.1) per 1,000. In comparison, the absolute risk following a negative LBC test was 9.3 (95% CI, 2.9 to 30.2). The relative sensitivity of AHPV compared to HC2 was 91.5% for CIN3+, and the negative predictive values were 99.8% (95% CI, 99.5 to 99.9%) for HC2 and 99.7% (95% CI, 99.4 to 99.8%) for AHPV. Our data show that the longitudinal performance of the AHPV test over six years is comparable to the performance of the HC2 test and that the absolute risk of CIN3+ over six years following a negative AHPV result in a screening population is low. (This study is registered at ClinicalTrials.gov under registration number NCT02634190.)

## Abstract (precis)

Longitudinal data on the E6/E7 mRNA-based Aptima human papillomavirus (AHPV) assay exceeding three years in comparison to the gold standard Digene Hybrid Capture 2 (HC2) test are not available. We previously reported the cross-sectional data of the German AHPV Screening Trial (GAST) in which 10,040 women were recruited and tested by liquid-based cytology, the HC2 assay, and the AHPV assay.

## INTRODUCTION

Systematic screening has led to a significant decrease in cervical cancer cases worldwide. Since persistent human papillomavirus (HPV) infection with 13 high-risk HPV (HR HPV) types defined as class I or IIA carcinogenic for women (1) is a necessary prerequisite for the development of precancerous lesions and cervical neoplasia, tests for HR HPV infections have been developed and validated. The incorporation of molecular HPV testing into cervical cancer screening programs results in fewer cases of cancer and high-grade cervical intraepithelial neoplasia (CIN3) being detected at the second screening round in those who were tested by HPV tests than in women screened by cytology only (2–8). The lower risk following HPV testing suggests that extended screening intervals are appropriate (9–11), which also avoids the detection of transient infections in consecutive screening rounds that leads to overtreatment.

Currently, more than 190 HPV assays are commercially available (12), and many countries have implemented HPV tests in their cervical cancer screening programs, while other countries are in the process of switching from cytological screening to primary HPV testing or cotesting (9, 13, 14), and national cervical cancer screening guidelines have been adapted accordingly (13). Five group tests are approved by the FDA for application in the United States and primarily detect the HR HPV group. To date, only the cobas 4800 HPV test (Roche, USA) and the Onclarity HPV assay (Becton, Dickinson) have been approved for first-line primary screening. The Digene Hybrid Capture 2 (HC2) high-risk HPV DNA test (Qiagen, Hilden, Germany) is considered the gold standard of HPV assays, as its performance was validated in a large number of randomized controlled trials, and it was the first HPV test to receive FDA approval to screen patients with atypical cells of undetermined signiﬁcance (ASCUS); also, in women 30 years and older, the HC2 high-risk HPV DNA test can be used with Pap to adjunctively screen to assess the presence or absence of high-risk HPV types. HC2 HPV detection is based on full-length genomic RNA probes for hybridization with the viral DNA of the 13 high-risk HPV types. Longitudinal data showed a very low risk of developing cervical cancer over at least 5 years in women with a negative HC2 baseline test (15). Four randomized trials have demonstrated that the cumulative incidence of cervical cancer after a median of 6.5 years after a negative HC2 test was lower than the cumulative incidence 3 years after a normal cytology result (10).

However, cross-hybridization of the HC2 test with at least 26 additional HPV types of low carcinogenicity or undefined risk has been detected (16–19), which occasionally can be found even in CIN3 (20). Epidemiological data suggest that such lesions are unlikely to progress to cervical cancer. This makes the use of HC2 as comparator test according to established consensus guidelines questionable, as CIN3 associated with non-HR HPV types (and of low progressive potential) will be detected by the HC2 test but not by a test with a more stringent analytical specificity that detects predominantly class I/IIa carcinogenic types (21).

As stated by Meijer et al., new HPV tests should demonstrate noninferior sensitivity and specificity compared with the HC2 test in a representative set of samples from a routine screening population in a cross-sectional setting (22). However, these guidelines specify that they apply to DNA-based tests, and whether this guideline could also be applied to the validation of RNA-based tests is controversial. A key issue is the definition of a representative sample (of cases of CIN2+) and which parameters are required to allow for the extension of screening intervals. It is argued that RNA positivity is a later event in the natural history of cervical neoplasia than HPV DNA positivity; hence, there is a desire to have more longitudinal data regarding CIN2+ incidence following a negative RNA-based test to determine whether it is safe to extend the screening interval after a negative RNA-based test.

The Aptima human papillomavirus (AHPV) assay (Hologic, San Diego, CA, USA) is based on target-mediated amplification for the detection of viral mRNA. The test detects the mRNA of the two HPV oncogenes E6 and E7 of 14 HPV types, which include all high-risk HPV types targeted by the HC2 assay as well as the class 2B type HPV66 (1, 23).

The RNA-based AHPV test has been compared to the DNA-based HPV tests in a number of studies, several of which are typical screening populations (23, 24). In these studies, the AHPV test consistently demonstrated comparable sensitivities for the detection of CIN2+ or CIN3+, as well as superior specificity.

We recently reported the cross-sectional results of the German AHPV Screening Trial (GAST), where the clinical sensitivities and specificities of the AHPV and the HC2 HPV tests were determined and compared in cervical samples from 9,451 women age 30 to 60 years from a routine screening population (24). Samples were centrally analyzed by liquid-based cytology (LBC), the AHPV assay, and the HC2 assay, and those women who had a positive result by any of these tests were referred for colposcopy. There was no statistical difference between the AHPV and the HC2 tests regarding their sensitivities in detecting CIN2 or CIN3+ lesions. The specificity (<CIN2) and the positive predictive value (CIN2+) of the AHPV test were significantly improved compared to the HC2 test. The GAST study results are in line with those from a previous report by Heideman et al., which confirmed the noninferiority of the AHPV assay versus the GP5+/6+ test and showed that the AHPV test fulfills cross-sectional clinical HPV test requirements for cervical screening (25). Recently, the longitudinal clinical performance of the AHPV assay compared to the HC2 test was analyzed in a prospective clinical study (CLEAR) that included 3 years of follow-up in 6,201 women (26). The estimated sensitivity of the AHPV test was similar and specificity slightly higher than those of the HC2 test. After 3 years of follow-up, women who were HPV negative (AHPV or HC2) at baseline had a very low risk of CIN2+ and CIN3+.

However, longitudinal data from a screening population cohort on the AHPV assay exceeding 3 years compared to the gold standard HC2 testing are important for reassurance, especially after the recent introduction of extended screening intervals (≥5 years) in some national cervical cancer screening programs. To address this lack of data, the GAST was continued by annually inviting all untreated women who remained positive by at least one of the three tests for follow-up screening. Furthermore, a randomly selected group of 4,000 women who were triple negative at baseline were invited for a second screening round after a mean of 6 years. We report here the first longitudinal data of more than 3 years regarding cumulative risk for CIN2/3+, clinical sensitivity, and negative predictive value (NPV) for the detection of histologically reviewed high-grade CIN by the RNA-based AHPV assay in comparison to the HC2 test (registered at ClinicalTrials.gov under registration no. NCT02634190).

## MATERIALS AND METHODS

### Participants.

Women age 30 to 60 years from the routine cervical cancer screening population of three German centers in Tübingen, Saarbrücken, and Freiburg were invited to participate in GAST. The data of the baseline cross-sectional study have previously been published (24). Written informed consent was obtained from each participant, and the study protocol was approved by all relevant ethics committees (Ethik-Kommission Universitätsklinikum Tübingen, reference no. 475/2008MPG1; Ethik-Kommission Alfred Ludwigs-Universität Freiburg, reference no. EK Freiburg 63/09; EthikKommission Landesärztekammer Baden-Württemberg, reference no. B-2009-030f; and Ethik-Kommission Ärztekammer des Saarlandes, reference no. 02/10).

### Study design.

The design of the baseline cross-sectional study was described previously (24). In brief, eligible consenting women (*n* = 10,040) had single liquid-based cytology samples (PreservCyt; Hologic, USA) taken during the annual routine gynecological examination. Liquid-based cytology (LBC; ThinPrep Pap test; Hologic), the Digene Hybrid Capture 2 (HC2) high-risk HPV DNA test, and the (AHPV) assay were performed on all samples. All women with a positive result in any of the three screening tests were referred for colposcopy within 8 weeks of receiving their test results.

For the positive follow-up arm of the GAST, all women who tested positive by any of these assays and who were not treated because of abnormal colposcopy and/or histology were retested annually for up to 5 years. After a mean interval of 6 years, 4,000 women who were triple negative at baseline were randomly selected and invited to be retested by all three tests when attending routine cervical screening (i.e., second screening round). Those who tested positive by any of the three tests were referred to colposcopy. The rationale for the sample size among women who tested triple negative at baseline can be found in the Supplemental Methods in the supplemental material.

### Liquid-based cytology.

As previously described for the cross-sectional trial (24), LBC results were evaluated according to the Munich nomenclature II and translated into the Bethesda System (TBS). LBC results were considered negative when the result was Pap I/II (equivalent to negative for intraepithelial lesion or malignancy [NILM]) or Pap IIw (equivalent to inadequate or atypical cells of undetermined signiﬁcance [ASCUS]); all other results were considered positive.

### HPV testing.

HPV testing was performed as previously detailed (24). Residual LBC samples were processed for HPV testing, according to the manufacturer’s speciﬁcations. The remaining samples were stored for INNO-LiPA Extra genotyping in case of positive HPV test results.

Digene Hybrid Capture 2 high-risk HPV DNA testing was performed as described previously (27), using the Rapid Capture system (RCS; Qiagen, Hilden, Germany), according to the instructions. A relative light units/cutoff (RLU/CO) ratio of 1.0 for positive test results was used as a cutoff in this study. All PreservCyt samples with an initial result of ≥1 and <2.5 RLU/CO were retested as recommended by the manufacturer. If the retest result was ≥1 RLU/CO, the final result was reported as positive. However, if the retest result was negative, a third test was performed to generate a final two out of three results.

The AHPV assay was performed following the manufacturer’s instructions. The earlier cutoff value of a signal/cutoff (S/CO) ratio of 1.0 instead of the current cutoff (0.5) was used throughout this study to provide continuity of the data. HPV genotyping was carried out using the INNO-LiPA HPV Extra genotyping test (Fujirebio, Ghent, Belgium), as described previously (27, 28).

### Disease ascertainment and histopathology.

Women who tested positive in either LBC, the AHPV assay, or the HC2 assay (HPV-positive women) were referred to colposcopy within 8 weeks. If lesions were detected after the application of acetic acid, a biopsy sample was taken from the suspicious tissue, and specimens were processed to produce hematoxylin and eosin (H&E)-stained slides. Current practice in Germany and some other European countries is to observe CIN2 lesions instead of treating them immediately, depending on the individual situation of the patient and her agreement. After local pathologist review, all slides were classified using the three-tiered CIN terminology. All slides with abnormal findings were reviewed by a second pathologist blinded to the first diagnosis, and slides with discordant review results were again reviewed by a third pathologist to reach a consensus diagnosis (two out of three agreement).

### Statistical analyses.

Prior to analysis, data were checked for plausibility and monitored. This included violation of inclusion criteria (pregnancy or age below 30 years or above 60 years), and positive Pap test six months prior to baseline testing as well as HIV infection. Following this, the databases were sealed and sent to the statisticians for statistical analysis.

Women with at least one positive test at baseline, who had at least one adequate screening test result on follow-up, and who were not treated nor diagnosed with CIN3 or worse at baseline were eligible for the follow-up analysis. In addition, women who tested negative at all three tests at baseline and had at least one adequate screening test result during follow-up were eligible for analysis.

We present baseline demographic characteristics from all participants in the study and for those who attended follow-up. To assess whether there was a statistical difference between groups, we used a chi-square test to compare those attending follow-up with those who were eligible to attend but did not.

### Estimating the cumulative risk of CIN3+ and CIN2+.

The follow-up of women in whom all three baseline screening tests were negative was quite different from that in women who had one or more positive screening test results at baseline. Those with all negative results were rescreened once after approximately 6 years. Women who had a positive result at baseline were invited back at 12- to 18-month intervals until the results of all three tests were negative or until they were treated for high-grade CIN.

At each visit, we estimated the hazard of having CIN3+ (or CIN2+) by multiplying the proportion of tested women who were eligible for colposcopy by the proportion of women attending colposcopy who were diagnosed with CIN3+ (or CIN2+). Further details can be found in the Supplemental Methods. Having estimated the hazard at each visit, we estimated the cumulative probability of disease after several visits using the Kaplan-Meier (product limit estimator) approach. The variance of the modified Kaplan-Meier estimator was derived in the same way as the Greenwood formula is derived for the usual Kaplan-Meier estimator. The formula is provided in the Supplemental Methods.

We estimated the hazard at baseline separately for each of eight groups based on the result combinations (positive or negative) for each of the three screening test results. Subsequently, we estimated the hazards separately in just four groups based on the baseline result combinations of the two HPV tests. There was no evidence that the hazards differed depending on the LBC result within each of the four groups (and numbers were too small to estimate hazards separately in each of the eight groups). Since we did not have 6-year follow-up data for women who were not triple negative at baseline, we assumed that the hazard observed at about 6 years in the baseline screen-negative group also applied to all other groups.

We then estimated the number of cases of CIN3+ (and CIN2+) that would have been observed among women negative at baseline by each of the three tests (separately) had everyone been followed to 6 years by taking a weighted sum of the estimated cumulative risk in each group. The cumulative risk in each group was also estimated by dividing the number of cases by the number of women with that result at baseline.

Confidence intervals were obtained by assuming that the logarithm of the cumulative risk is approximately normally distributed. *P* values were estimated from the discordant pairs using the exact McNemar significance probability test.

The main analysis presents results that included all CIN3 cases. Since we were interested in comparing the performances of two HPV tests, both of which aim to detect the same 13 high-risk HPV types and HPV66, and since CIN3 caused by other HPV types is less likely to progress to cancer, a subanalysis excludes disease where the HC2 results are technically false positive due to cross-hybridization with noncarcinogenic HPV types (i.e., we include only lesions positive for one of the 13 types classified as class I/IIa carcinogenic to humans).

Analyses were carried out using STATA 15.0 (StataCorp).

## RESULTS

**FIG 1:**
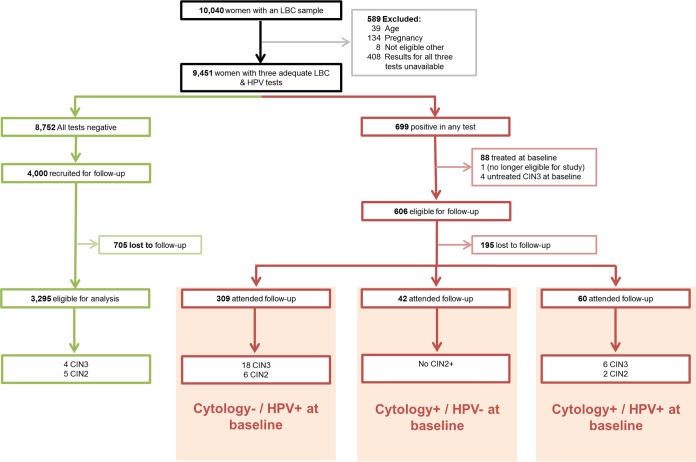
Follow-up flow chart. Green indicates the cohort which tested triple negative (LBC, HC2, and AHPV) at baseline. Red indicates the cohort who tested positive by at least one test (LBC, HC2, or AHPV) at baseline.

Out of the 4,000 women with negative screening test results at baseline who were invited, 3,295 women (82.4%) attended follow-up. Among those with at least one positive test at baseline, 606 women were eligible for follow-up, and 411 women (67.8%) attended (Fig. 1). The baseline demographic characteristics of women eligible for analysis at baseline and follow-up are presented in Table 1. Women who participated in the cross-sectional study were broadly similar to those who attended follow-up. There were only slight differences in education and the number of sexual partners between women attending follow-up and those who were eligible but who did not attend.

**TABLE 1:** Baseline demographic characteristics of women in GAST

Characteristic reported at baseline	Attended follow-up		Eligible for analysis at baseline		Chi-square test^*a*^
	*n*	%	*n*	%	
Age at enrollment (yr)					
30–34	611	16.5	1,623	17.2	
35–39	692	18.7	1,696	17.9	
40–44	852	23.0	2,123	22.5	
45–49	734	19.8	1,873	19.8	
50–54	499	13.5	1,295	13.7	
55–59	318	8.6	841	8.9	
Missing data	0		0		
Total (not missing)	3,706		9,451		*x*^2^_5_ = 5.163, *P* = 0.396
Education					
Missing data	384		1,663		
None	13	0.4	34	0.4	
Primary	602	18.1	1,383	17.8	
College	1,503	45.2	3,350	43.0	
University	1,204	36.2	3,021	38.8	
Total (not missing)	3,322		7,788		*x*^2^_3 _= 17.16, *P* = 0.001
No. of sexual partners					
Missing data	1,016		3,107		
One	921	34.2	2,195	34.6	
Two to four	1,019	37.9	2,282	36.0	
Four or more	750	27.9	1,867	29.4	
Total (not missing)	2,690		6,344		*x*^2^_2_ = 8.6423, *P* = 0.013
Age at first sexual intercourse (yr)					
Missing data	737		2,448		
Under age 18	1,660	55.9	3,855	55.0	
Age 18 or older	1,310	44.1	3,148	45.0	
Total (not missing)	2,970		7,003		*x*^2^_1_ = 1.4558, *P* = 0.228

a Note the *x*^2^ test compares those attending to those not attending among those eligible for follow-up. Subscript numbers represent degrees of freedom in each chi-square test.

### Results for women on follow-up after a positive baseline test result.

Untreated women with at least one positive test result at baseline and no CIN3 or worse were invited to attend annual follow-up examinations over a 5 year-period (*n* = 606). Follow-up ceased when HPV infection and/or cervical abnormalities were cleared, if treated for cervical disease, or if they refused to participate in the follow-up study. Of the eligible women, 411 (67.8%) attended at least one follow-up examination and were eligible for analysis. The median time to the first follow-up visit was 14 months (range, 6 to 80 months) (see Fig. S1 in the supplemental material), and the average number of follow-up visits per participant was 1.7 (range, 1 to 5). Three women were excluded because they were missing an HC2 test result and did not return for follow-up. Of the 408 women with at least one follow-up visit with adequate HC2 and AHPV results, 77.2% (315) were negative on both HPV tests at their final visit. In total, 200 women tested positive during follow-up and were referred to colposcopy; 165 (82.5%) of these women attended colposcopy. Ninety percent of those who attended follow-up did so within 2.5 years of baseline. A total of 32 women were diagnosed with CIN2 or worse during follow-up.

The follow-up HPV test results by visit number among those with a positive screening test at baseline are detailed in Table S1. No LBC test results were missing, but 10 women had both HPV test results missing, and 19 women were missing the HC2 test result on at least one appointment. The agreement of the HPV tests (when both were available) was substantial, with a kappa (κ) value of 74.7% (95% confidence interval [95% CI], 69.7% to 79.7%).

During follow-up of those women who tested positive on at least one test at baseline, a total of 24 women were diagnosed with CIN3 and 8 women with CIN2 (Table 2). The baseline test results and numbers diagnosed with CIN2+ during follow-up are shown in Table 2. Twenty-four CIN2+ lesions (75%) were detected in women with negative cytology and with at least one positive HPV test result and 8 lesions (25%) in women who tested triple positive at baseline. At baseline, the HC2 result was negative in one CIN2 and two CIN3 cases, while the AHPV result was negative in one CIN2 and five CIN3 cases that developed during follow-up (data not shown). One of the 5 CIN3 cases with discordant HR HC2-positive and AHPV-negative HPV test results at baseline was identified by genotyping as HPV82, which is not targeted by either assay and which is not an HR type. No adenocarcinoma *in situ* (AIS) or invasive cervical cancer cases were detected during follow-up.

**TABLE 2:** Baseline HPV test results among women with CIN2+ during follow-up

LBC result	HC2 result	AHPV result	No. with CIN2	No. with CIN3
+	+	+	2	6
−	+	+	4	11
−	+	−	1	5^*a*^
−	−	+	1	2

a One case was positive for HPV82 on the INNO-LiPA and is excluded in the subanalysis.

### Longitudinal results for women who tested negative at baseline.

In the baseline cross-sectional arm of the German AHPV Screening Trial (GAST), 8,752 women had a negative result in all three tests (cytological screening, Hybrid Capture 2 [HC2], and AHPV). Of these, 4,000 participants were invited for follow-up testing approximately 6 years postenrollment. In total, 3,295 participants (82.4%) attended follow-up (Fig. 1). The median time between baseline and attendance at the second round was 6.2 years (range, 3.9 to 8.5 years).

At the second round, 3,057 women tested negative on all three tests (92.8%). A total of 140 women (4.6%) had at least one positive test results at follow-up, 115 (82%) of these women underwent a colposcopic examination, and a total of 9 women were diagnosed with CIN2 or worse disease (5 CIN2 and 4 CIN3 lesions). A summary of the LBC and HPV test results at the second screening round is found in Table 3. The level of agreement between the HPV tests was substantial, with a kappa value of 81.1 (95% CI, 78.0 to 93.8).

**TABLE 3:** Second-round LBC and HPV test results among women who were triple negative at baseline

HPV test result during follow-up (HC2/AHPV)	LBC results (no.)				Total no.	No. with CIN2+
	Negative	Inadequate	Low-grade (Pap III)	High-grade (Pap IIID)		
Both missing	71	4	0	0	75	0
Missing HC2 result	1	0	0	0	1	0
Missing AHPV result	4	0	0	0	4	0
−/−	3,057	18	5	12	3,092	0
−/+	13	0	0	0	13	0
+/−	48	0	1	1	50	1
+/+	44	0	3	13	60	8
Total	3,238	22	9	26	3,295	9

The sensitivity of cytology for the detection of CIN3+ was 44% (*n* = 4 of 9), but 100% tested HPV positive (Table 4). One CIN3 case which tested HC2 positive and AHPV negative revealed in the histopathology a small lesion of 0.2 mm that was regressive and showed signs of inflammation. HPV16 was detected in all patients with CIN3 by the INNO-LiPA Extra genotyping test.

**TABLE 4:** Second-round screening HPV test results among women with CIN2+ during follow-up

LBC result	HC2 result	AHPV result	No. with CIN2	No. with CIN3+^*a*^
+	+	+	3	1
−	+	+	2	2
−	+	−	0	1

a Note the one CIN3+ with discordant HPV test results was also negative by LBC, as were two other CIN3 and two CIN2 results.

In the present study, we observed 10 of 23 untreated (43%) CIN2 cases that regressed, while 3/23 (13%) progressed to CIN3.

Passive clinical follow-up data were available from a registry on the complete Saarbrücken subcohort of 2,147 women who tested triple negative at baseline; 887 of those women attended follow-up as part of GAST. During a 6-year passive follow-up period, only one CIN1 and one CIN2 case were observed in women who did not attend the second-round screening in GAST. Among the Saarbrücken cohort attending the second screening round, one case of CIN2 and two cases of CIN3 were detected at the second screening appointment.

### HPV types in samples with high-grade disease.

All HPV-positive samples were genotyped by the INNO-LiPA Extra genotyping test. Baseline HPV test results among the 41 women who went on to be diagnosed with CIN2+ during follow-up show that 9 women (22%) were HPV negative, 2 women (5%) tested positive for HC2 non-high-risk HPV types (HPV66 and HPV82), and 24 (58%) single-type and 8 (20%) multiple-type HPV infections were detected (results not shown).

HPV genotyping results at the time of diagnosis (during follow-up) are presented in Table S2. At the time of diagnosis, 1/41 (2%) CIN2 case was HPV negative on both tests, one CIN2 case and one CIN3 case (5%) tested positive to non-high-risk HPV types (HPV53, HPV66, and HPV82), and 28/41 (68%) single-type and 12/41 (29%) multiple-type HPV infections were detected.

HPV16 was the most frequent HPV type detected in patients with CIN3 in the cross-sectional part of the study and among those attending follow-up.

### Cumulative risk of disease during the study period.

The main analysis presents results that include all diagnosed disease. We present a subanalysis excluding two CIN3 cases whose HPV types were HPV82 and HPV67 and hence were considered technically false-positive HC2 test results. One case (HPV67) was diagnosed and treated at baseline, and the remaining case (HPV82) was diagnosed at follow-up.

**FIG 2:**
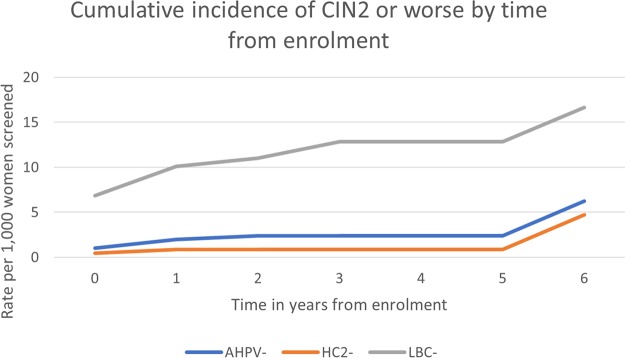
Rate of CIN2 or worse per 1,000 women screened following a negative baseline test result. Follow-up visits should have been annual up to 5 years for those with a positive test result at baseline and at 6 years for those with triple negative baseline test results.

**FIG 3:**
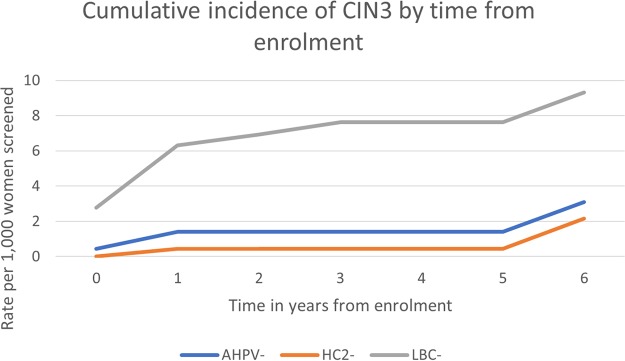
Rate of CIN3+ per 1,000 women screened following a negative baseline test result. Follow-up visits should have been annual up to 5 years for those with a positive test result at baseline and at 6 years for those with triple negative baseline test results.

A summary of the 6-year cumulative risk per 1,000 women screened and negative predictive value among women testing negative at baseline can be found in Table 5. The absolute risk per 1,000 women screened by time since a baseline test is presented in Fig. 2 and 3. Note that the vast majority of women who were negative by any one screening test were negative by all three tests and were therefore not rescreened until 6 years. This explains the sudden jump in the risk at year 6 visits.

**TABLE 5:** Six-year cumulative incidence, risk per 1,000 women screened, and negative predictive value among those testing negative at baseline

Characteristic	Cumulative incidence (% [95% CI])		Risk per 1,000 women screened (95% CI)		Negative predictive value(% [95% CI])^*a*^	
CIN2 or worse						
AHPV negative	0.62	0.24–1.59	6.2	2.4–15.9	99.38	98.41–99.76
HC2 negative	0.47	0.27–0.81	4.7	2.7–8.1	99.53	99.19–99.73
LBC negative	1.66	0.72–3.83	16.6	7.2–38.3	98.34	96.17–99.28
CIN3 or worse						
AHPV negative	0.31	0.17–0.57	3.1	1.7–5.7	99.69	99.43–99.83
HC2 negative	0.22	0.10–0.49	2.2	1.0–4.9	99.78	99.51–99.90
LBC negative	0.93	0.29–3.02	9.3	2.9–30.2	99.07	96.98–99.71

a Note the NPV is estimated excluding the risk among those attending the second round of screening.

### CIN2 or worse.

The cumulative risks of CIN2 or worse by the year 6 visit were 0.62% (95% CI, 0.24% to 1.59%) and 0.47% (95% CI, 0.27% to 0.81%) among those who tested AHPV and HC2 negative, respectively. The difference in AHPV-negative cases was 0.15% (95% CI, 0.38% less to 0.69% more) and is not significant (*P* = 0.096). For comparison, the cumulative risk by 6 years among LBC-negative women was 1.66% (95% CI, 0.72% to 3.83%). The relative sensitivity for CIN2+ of the AHPV test in comparison to the HC2 test was 91.4%. Among women testing negative by both HPV tests at baseline, the cumulative risk of CIN2 or worse was 0.38% (95% CI, 0.17% to 0.86%).

The subanalysis excluding one case (diagnosed at follow-up) of CIN3 that tested HPV82 positive and one (diagnosed at baseline) that tested HPV67 positive produced very similar results, with cumulative risks at 6 years of 0.59% (95% CI, 0.22% to 1.61%) and 0.47% (95% CI, 0.27% to 0.81%) among AHPV- and HC2-negative women, respectively. The relative sensitivity for CIN2+ of AHPV in comparison to HC2 was 93.0%.

### CIN3.

The cumulative risks of CIN3 disease by the year 6 visit were 0.31% (95% CI, 0.17% to 0.57%) and 0.22% (95% CI, 0.10% to 0.49%) for AHPV-negative and HC2-negative women (Table 5), respectively (difference, 0.09% [95% CI, −0.02% to 0.21%]). The cumulative risk by the year 6 visit among those testing LBC negative was 0.93% (0.29% to 3.02%). The relative sensitivity for CIN3 of the AHPV test in comparison to HC2 was 91.5%. Among women testing negative by both HPV tests at baseline, the cumulative risk of CIN3 was 0.17% (95% CI, 0.04% to 0.75%).

The subanalysis excluding two CIN3 cases with technically false-positive HR HC2 HPV type results produced cumulative risks by 6 years of 0.28% (95% CI, 0.14% to 0.54%) among those who tested AHPV negative at baseline and 0.22% (95% CI, 0.10% to 0.49%) among those who tested HC2 negative (*P* = 0.1094). The cumulative risk by 6 years among those testing LBC negative was 0.90% (95% CI, 0.27% to 3.04%). The relative sensitivity of the AHPV test to the HC2 test for CIN3 increased to 94.2%.

There were only 20 women with a signal/cutoff ratio of between 0.5 and 1.0 on the AHPV test at baseline, and they were all HC2 negative and LBC negative. Only four of these 20 women attended follow-up, where they were found to still be HPV negative.

## DISCUSSION

In recent years, many countries have integrated HPV testing into their national cervical cancer screening programs. Compared to conventional methods, HPV testing increases early detection rates of precancerous and cancerous lesions and allows extended screening intervals. However, the optimal lengths of screening intervals for women with negative results remain to be established and might greatly depend on the long-term predictive values of a given HPV test. Longitudinal clinical performance data have so far been published for only a small number of HPV tests. Ronco et al. presented pooled data from four studies on the performance of the DNA-based HC2 assay over a median 6.5-year follow-up period (10). In addition, there is evidence regarding the good negative predictive value over 3 years for the cobas 4800 test (Roche Diagnostics) (29), over 3 years for the Abbott RealTime HPV DNA test (30), and over 3 years for the RNA-based AHPV test (26). During the revision of our manuscript, data comparing the AHPV test with the cobas 4800 HPV test using biobanked material were published that demonstrate a noninferior longitudinal sensitivity and NPV over 7 years for the AHPV test (31).

In the present study, we evaluated the extended predictive value of the RNA-based AHPV test in comparison to the DNA-based HC2 test over a 5- to 6-year period by focusing on the cumulative risks for CIN3+ 6 years after a negative baseline result. In our opinion, CIN2+ is a less reliable endpoint because it is an equivocal histological diagnosis, and regression rates are high, as observed in our study, at 43%. An advantage of this study, therefore, was that many CIN2 lesions were not treated immediately and were seen to regress during surveillance.

During the course of the follow-up of women who tested positive (LBC, AHPV, or HC2) at baseline, we detected 8 CIN2 and 24 CIN3 cases. One CIN2 case was missed by both HPV tests and was positive only by cytology. One CIN3 case tested negative by the AHPV test at the time of diagnosis but was detected by the HC2 test, and it contained a non-HR type (HPV82). Results from a meta-analysis of type-specific HPV DNA prevalence in cervical cancer and women with normal cytology showed prevalences of HPV82 of 0.1% (95% CI, 0.1 to 0.3%) and 0.1% (95% CI, 0.0 to 0.1%), respectively. HPV82 is not targeted by the HC2 test but may yield positive results due to a cross-reaction. The known extensive cross-reactivity of the HC2 test may therefore explain the nonsignificantly higher sensitivity than that of the AHPV test in the baseline and follow-up results of this study. According to the Meijer et al. criteria (22), the candidate test should have a clinical sensitivity for CIN2+ not lower than 90% of the clinical sensitivity of the HC2 in women age at least 30 years. Clearly, the results for the AHPV test at 6 years achieved clinical sensitivity rates exceeding this 90% threshold, regardless of if all CIN2+ cases were included or if CIN2+ with noncarcinogenic types were excluded.

In the second screening round of women who tested triple negative at baseline, a total of five CIN2 and four CIN3 cases were identified, of which one CIN3 was missed by the AHPV test at follow-up. This case was HPV16 positive and was repeatedly detected positive by the HC2 test at 1.86, 1.53 and 1.51 RLU, which is a borderline positive result according to the FDA approval but a negative test result in some countries (e.g., United Kingdom), where an increased cutoff of 2.0 RLU is used for cervical cancer screening.

The cumulative risks of CIN3 + 6 years after a negative screening test in this study are very similar to the ones observed earlier by Dillner et al. (15). In both studies, the cumulative incidence in women with baseline negative cytology was around 1%. The cumulative incidence after a negative AHPV test result in this study was substantially lower, at 0.31% (0.17% to 0.57%). This is in line with one previous publication where very low 3-year risks for CIN3+ were detected after a negative baseline AHPV test (26). In our study, the upper 95% confidence limit for the additional risk after a negative AHPV compared with a negative HC2 result was 0.21%. If it is accepted that women do not need to be rescreened until their risk of CIN3+ reaches 0.5%, this study has shown that it is safe to use an interval of 5 to 6 years after a negative AHPV test.

The analysis including all CIN3 cases detected and the analysis excluding two CIN3 cases (one from baseline with HPV67 and one detected at follow up with HPV82) revealed highly comparable absolute risks for CIN3+ following a negative baseline HC2 or AHPV test and comparable longitudinal negative predictive values (NPV) for the tests. Note that women who tested positive (by any test) at baseline were not asked to return for testing 6 years after enrollment. Therefore, we assumed that the hazard at 6 years among those who tested positive but who did not develop disease was the same as that observed at about 6 years in the baseline screen negative group. This explains the sudden jump in the risk shown in Fig. 2 and 3 after 5 years.

Altogether, we found an absolute risk for developing CIN3+ after 6 years among those women who tested negative at baseline of 2.2 and 3.1 per 1,000 women screened by the HC2 and the AHPV test, respectively. This difference is not significant and is in line with one previous publication where very low 3-year risks for CIN3+ were detected after a negative baseline AHPV test (26).

The fact that annual follow-up of all women from the routine screening population was unachievable complicated the data analysis and might be considered a weakness of this study. However, the CLEAR study found that annual follow-up of screen-negative women suffers from low compliance (26) and is not recommended by national and European guidelines. Passive follow-up of those women who tested triple negative at baseline was possible in a subset of 2,147/8,752 (25%) women and showed that we were unlikely to have missed disease by only rescreening at 6 years. On the other hand, this study strongly benefits from the large number of participating women, the prospective study design, and the extended follow-up period of 6 years. Another strength of this study is that all positive samples (LBC, AHPV, or HC2) were genotyped, which enabled a detailed analysis of discordant test results.

The poor sensitivity of LBC in this study may be considered a weakness but simply reflects the low single-round sensitivity of cytology in Germany that has been noted in several studies previously (32). Since the primary comparison is between the AHPV and HC2 tests, the poor sensitivity of LBC does not affect the overall results.

In summary, numerous studies from different populations (23) consistently demonstrated a similar cross-sectional sensitivity paired with higher clinical specificity when AHPV was compared to other FDA-approved HPV DNA tests, which reduces the costs of follow-up. With regard to the extended intervals in some cervical cancer screening programs, data for screening intervals up to 3 years have already been published, as well as a retrospective analysis over 7 years (31). With the present study, we add prospective data of the longitudinal performance over a 5- to 6-year period showing that the cumulative risk for CIN2/3 and the NPV of the AHPV test is nonsignificantly different from the HC2 assay. We conclusively demonstrate low absolute risks for CIN3+ following a negative AHPV test result, suggesting that the extended screening intervals proposed for use with the HC2 test are safe with the AHPV test as well.

## Supplementary Material

Supplemental file 1
