# Supplementary material for: Longitudinal Clinical Performance of the RNA-Based Aptima Human Papillomavirus (AHPV) Assay in Comparison to the DNA-Based Hybrid Capture 2 HPV Test in Two Consecutive Screening Rounds with a 6-Year Interval in Germany
Source: J Clin Microbiol. 2019 Jan 2;57(1):e01177-18. doi: 10.1128/JCM.01177-18 (PMC6322477; doi:10.1128/JCM.01177-18)
Supplement: Supplemental file 1 [file d569337db692ac0f7a482d6c3f8200db_JCM.01177-18-s0001.pdf]

Supplemental Material

Supplemental Methods

Sample size rational for follow-up of those who tested triple negative at baseline

The proposed sample size of 4000 patients was motivated by the following considerations:

In the cross-sectional analysis we found a prevalence rate for CIN3+ of 0.4% among all women, including HC2-/ APTIMA-/LBC-positives and triple negatives (prevalence). We assumed that the incidence within the group of triple negatives was at most equal to the prevalence of 0.4% found in the cross-sectional study including test positives. This was a very conservative assumption as the cumulative incidence after 5 years was to be expected smaller than the prevalence and triple negatives should have a smaller incidence as the entire group. The assumption of 0.4% incident cases within triple negatives corresponds to an NPV (negative predictive value) of 99.6%. This NPV is used as the alternative in a statistical test. With n= 4000 a value of 99.3% could be rejected in a one-sided statistical test with a level of significance of 5% and a statistical power of 80%.

Estimating the cumulative risk of CIN3+ (and CIN2+): further details

Note that if a woman failed to attend colposcopy after that visit, but disease was found on the next visit, it was counted as if she had attended straight away and disease was found at that time. Similarly, if a woman failed to attend colposcopy but subsequently either had a negative colposcopy or an all-negative screen, it was counted as if she had attended colposcopy (and did not have disease) at the earlier visit. Everyone had a baseline visit. The rule we have used here moving some outcomes from visit 1 to baseline, means that the baseline results are not identical to those that have been published previously. (Iftner T, et

al. 2015. Head-to-Head Comparison of the RNA-Based Aptima Human Papillomavirus (HPV) Assay and the DNA-Based Hybrid Capture 2 HPV Test in a Routine Screening Population of Women Aged 30 to 60 Years in Germany. J Clin Microbiol .)

Formula for the variance of the modified Kaplan-Meier estimator

Let

N_i = **N**umber of women tested at visit i

R_i = Number of women **R**eferred to colposcopy at visit i

A_i = Number of women who **A**ttended colposcopy at visit i

C_i = Number of women with disease (**C**IN2+ or CIN3+) diagnosed at visit i.

Let $\Delta_i = \frac{R_i}{N_i}\frac{C_i}{A_i}$ be the risk of having disease if tested at visit i and $\ln S = \sum_{i=0}^{k} \ln(1-\Delta_i)$. Then

$$\operatorname{var} \ln S = \sum_{i=0}^{k} \frac{\left(\frac{R_i}{N_i}\right)^2 \frac{C_i}{A_i}\left(\frac{A_i - C_i}{A_i}\right)\frac{1}{A_i} + \left(\frac{C_i}{A_i}\right)^2 \frac{R_i}{N_i}\left(\frac{N_i - R_i}{N_i}\right)\frac{1}{N_i}}{(1-\Delta_i)^2}$$

$$= \sum_{i=0}^{k} \frac{\Delta_i}{N_i A_i} \frac{\left(\frac{R_i(A_i - C_i)}{A_i}\right) + \left(\frac{C_i(N_i - R_i)}{N_i}\right)}{(1-\Delta_i)^2}$$

$$= \sum_{i=0}^{k} \frac{R_i C_i}{(N_i A_i - R_i C_i)^2}\left(\frac{R_i(A_i - C_i)}{A_i} + \frac{C_i(N_i - R_i)}{N_i}\right)$$

Supplemental Figures

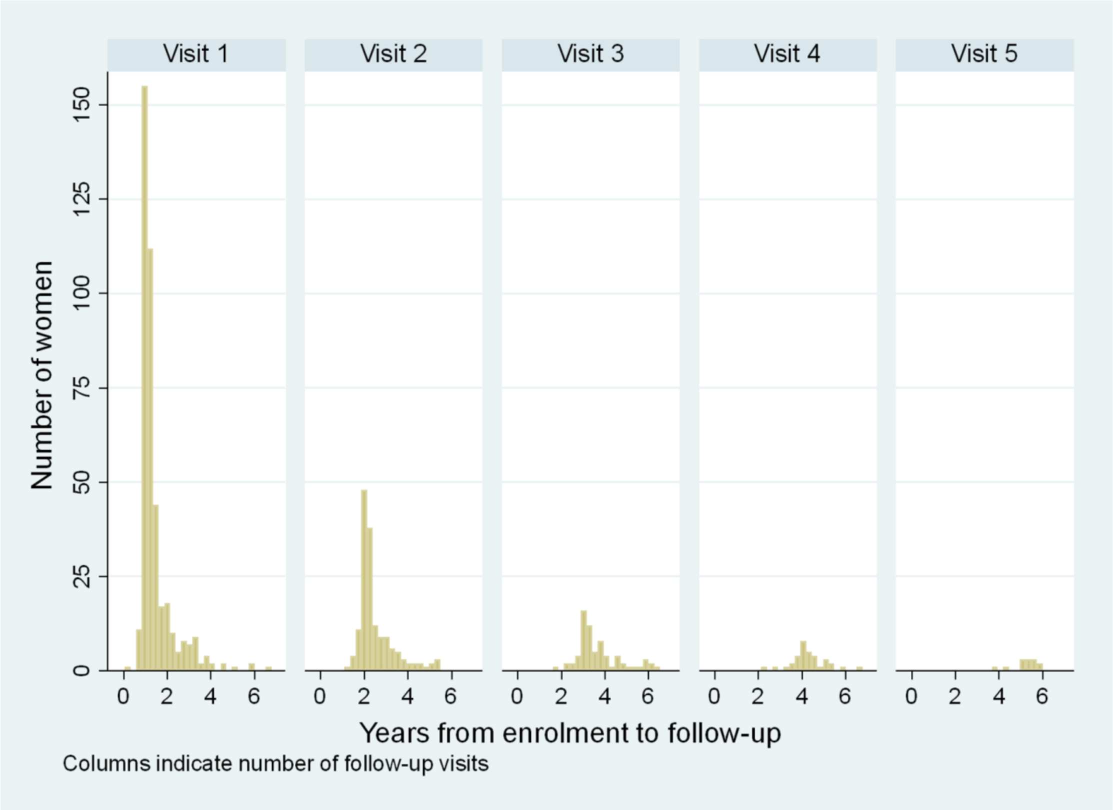

Figure S1: Time to follow-up

Supplemental Tables

Table S1. HPV test results among women attending follow-up following a positive screening test at baseline but no treatable disease

	HPV test result during follow-up (HC2/AHPV)							
Follow-up visit number	No HPV test	No HC2*	-/-	-/+	+/-	+/+	Total	CIN2+ (total)
One	5	11	223	15	31	126	411	23
Two	3	6	108	6	13	63	199	6
Three	0	2	23	2	9	35	71	1
Four	2	0	15	3	4	10	34	2
Five	0	0	8	0	2	3	13	0
CIN2+ (total)	0	0	1	1	1	29		32

*There were no visits where AHPV results were missing

Table S2. Test result and HPV genotyping upon follow-up among those with CIN2 or worse disease

HPV Type(at time of diagnosis)	Test results at time of diagnosis		
**CIN2 (negative at baseline)**	**LBC**	**HR HC2**	**AHPV**
56	+	+	+
39,51,58,56/74,6	+	+	+
53,66	+	+	+
18	-	+	+
16,31,6,69/71	-	+	+
**CIN3 (negative at baseline)**			
16	+	+	+
16	-	+	-
16	-	+	+
16	-	+	+
**CIN2 (positive at baseline)**			
18,31	+	+	+
16	-	+	+
16	-	-	+
*	+	-	-
31	-	+	+
52	+	+	+
16	+	+	+
45,56	+	+	+
**CIN3 (positive at baseline)**			
45,74	-	+	+
16	-	+	+
16,56	+	+	+
31	+	+	+
31	-	+	+
16	-	+	+
82	-	+	-
16	-	+	+
52	-	+	+
18	+	+	+
31	+	+	+
16,31	-	+	+
33,40,69/71	-	+	+
31	+	+	+
16,52,59	+	+	+
52	-	+	+

56	+	+	+
31	+	+	+
16	-	+	+
31	+	+	+
33	+	+	+
31,51	+	+	+
16,31	-	+	+
51	+	+	+

*at time of diagnosis no material was available for LiPA genotyping, at baseline and f-up1 HPV16 was detected
